# Ecological Niche Modeling Identifies Fine-Scale Areas at High Risk of Dengue Fever in the Pearl River Delta, China

**DOI:** 10.3390/ijerph14060619

**Published:** 2017-06-09

**Authors:** Qiaoxuan Li, Hongyan Ren, Lan Zheng, Wei Cao, An Zhang, Dafang Zhuang, Liang Lu, Huixian Jiang

**Affiliations:** 1 College of Geographical Sciences, Fujian Normal University, No. 8 Shangsan Road, Fuzhou 350007, China; liqx@lreis.ac.cn; 2 State Key Laboratory of Resources and Environmental Information System, Institute of Geographic Sciences and Natural Resources Research, Chinese Academy of Sciences, 11A Datun Road, Chaoyang District, Beijing 100101, China; zhenglan1007@163.com (L.Z.); caowei@igsnrr.ac.cn (W.C.); zhangan@igsnrr.ac.cn (A.Z.); zhuangdf@lreis.ac.cn (D.Z.); 3 Key Laboratory of Geographic Information Sciences, Ministry of Education, East China Normal University, No. 500 Dongchuan Road, Shanghai 200241, China; 4 State Key Laboratory for Infectious Diseases Prevention and Control, National Institute for Communicable Disease Control and Prevention, China CDC, No.5 Changbai Road, Changping District, Beijing 102206, China; luliang@icdc.cn

**Keywords:** dengue fever, Maxent, socioeconomic factors, environmental conditions, Guangzhou, Foshan

## Abstract

Dengue fever (DF) is one of the most common and rapidly spreading mosquito-borne viral diseases in tropical and subtropical regions. In recent years, this imported disease has posed a serious threat to public health in China, especially in the Pearl River Delta (PRD). Although the severity of DF outbreaks in the PRD is generally associated with known risk factors, fine scale assessments of areas at high risk for DF outbreaks are limited. We built five ecological niche models to identify such areas including a variety of climatic, environmental, and socioeconomic variables, as well as, in some models, extracted principal components. All the models we tested accurately identified the risk of DF, the area under the receiver operating characteristic curve (AUC) were greater than 0.8, but the model using all original variables was the most accurate (AUC = 0.906). Socioeconomic variables had a greater impact on this model (total contribution 55.27%) than climatic and environmental variables (total contribution 44.93%). We found the highest risk of DF outbreaks on the border of Guangzhou and Foshan (in the central PRD), and in northern Zhongshan (in the southern PRD). Our fine-scale results may help health agencies to focus epidemic monitoring tightly on the areas at highest risk of DF outbreaks.

## 1. Introduction

Dengue fever (DF) is an insect-borne disease caused by four different dengue viruses (DENV 1–4), which are mainly transmitted by *Ae. aegypti* and *Ae. Albopictus* [1]. DF is endemic to more than 100 countries in tropical and subtropical areas, especially in Southeast Asia, the Americas, the Western Pacific, Africa and the Eastern Mediterranean [2]. The overall incidence of DF has increased 30-fold in the past 50 years, with about 2.5 billion people living in DF-endemic risk areas [2].

There were no documented cases of DF in China after 1949 until an outbreak in Guangdong Province in 1978 [1]. Although DF in Mainland China continues to be seen as an imported infection, the prevalence of this disease has increased steadily over the past 15 years, especially in the Southern Chinese provinces of Guangdong, Zhejiang, Yunan, Fujian, and Guangxi [1,3,4,5]. In the last 11 years (2004 to 2014), more than 55,000 cases were reported in Mainland China. Approximately 94% of the local cases that emerged in this period were reported in Guangdong Province, and most of these cases (more than 90%) were located in the Pearl River Delta (PRD) [1,6,7,8].

Although all DF epidemics in the PRD have been triggered by imported cases, local climatic and environmental conditions such as precipitation, temperature and humidity determine the size of the outbreak [8,9]. The geographic distribution of DF cases in an outbreak is also related to socioeconomic factors such as population size, affluence, and access to public transportation [10,11,12,13,14,15]. A number of studies have demonstrated the relationship between the severity of an epidemic and various risk factors, and pointed out that it is effective to capture the impact of risk factors on the geographical distribution of the epidemic on a suitable spatial and temporal scale [16,17,18,19,20]. These studies have contributed important insights regarding the environmental and socioeconomic factors associated with DF in the PRD at a large scale (local to regional). However, understanding of the spatial distribution of epidemic risk at a finer scale and the relative contribution of different risk factors is still limited.

Thus, the main objectives of this study were to:(1) identify the fine-scale spatial pattern of areas in the PRD at high potential risk for DF outbreaks and (2) analyze the main factors that affect this spatial pattern. To achieve these aims, we will use an ecological niche model (ENM) integrating climatic, environmental and socioeconomic variables. Results of this study may prove useful in the prevention and control of DF in the PRD and southern China.

## 2. Materials and Methods

### 2.1. Study Area

The PRD is the low-lying area surrounding the Pearl River estuary, located in southern China, adjacent to Hong Kong and the Macao Special Administrative Region. It has an area of 44,700 square kilometers and a population of some 58.74 million (as of the end of 2015). This highly developed region contains nine densely populated cities, including Guangzhou, Foshan, and Shenzhen [21]. As one of the main transport hubs between Mainland China and abroad, the population in the PRD is largely transient.

**Figure 1:**
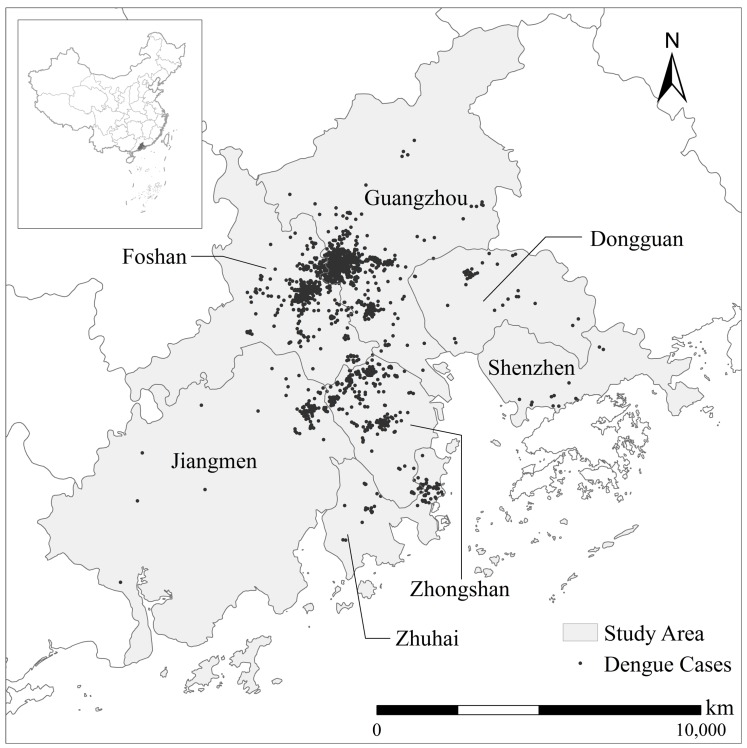
Recorded cases of dengue fever in the Pearl River Delta from 2003 to 2013.

The PRD also has a humid subtropical climate with hot, wet summers and mild, dry winters, ideal conditions for *Ae. aegypti* and *Ae. albopictus*, the primary vector of DF transmission in the PRD [4]. For the purposes of this study, we include only the seven cities in the PRD where DF cases have been most frequently recorded: Guangzhou, Shenzhen, Dongguan, Foshan, Zhongshan, Zhuhai and Jiangmen (Figure 1).

### 2.2. Data Collection and Preprocessing

#### 2.2.1. Dengue Cases

DF is a notifiable disease in China: diagnosed cases must be reported to the web-based National Notifiable Infectious Disease Reporting Information System within 24 h [6]. As the spatial pattern of a DF epidemic may vary in four to five year epidemic cycles [10,12,18], previous studies have minimized resultant biases by integrating seven to eight years of data [7,10,22,23]. We mimicked this approach by obtaining all DF case reports for the PRD over the 11-year period from 2003 to 2013 from the Chinese Center for Disease Control and Prevention (China CDC). We eliminated imported DF cases from our study, on the assumption that local cases better reflect the association between the pattern of the epidemic and local variables (environmental and social). The DF case-point data was obtained by geocoding (www.geocoding.cn) the addresses from each DF case report (Figure 1). There were a total of 4533 case points recorded between 2003 and 2013.

#### 2.2.2. Environmental Conditions

The climatic variables for the PRD were obtained from the National Meteorological Information Center of CMA, China. We interpolated average monthly climatic data (temperature, humidity, and precipitation) collected from weather stations between 2003 and 2013, and used the biological climate algorithm provided by WorldClim (http://www.worldclim.org/bioclim) to calculate new climatic variables. The normalized difference vegetation index (NDVI), measured by satellite remote sensors, isa simple graphical indicator that can be used to assess vegetation distribution and performance. NDVI values range from −1 to +1, where high values correspond to lush vegetation and low values correspond to an absence of vegetation [24]. Monthly NDVI data for the PRD, obtained from the Level-1 and Atmosphere Archive and Distribution System Web Interface (LAADS DAAC, https://ladsweb.nascom.nasa.gov), were recalculated into mean value of two quarters (warmest and coldest). River density, the total length of natural and artificial river channels per square kilometer of land, was obtained from the Data Center of Resources and Environmental Science, China (RESDC, http://www.resdc.cn).

#### 2.2.3. Socioeconomic Factors

Four socioeconomic variables were obtained from RESDC to represent the socioeconomic factors. Land Use and Land Cover Change (LUCC) data, the only categorical variable, was reclassified into five classes (developed, wetlands, agriculture, grassland, and forest) to represent different land use in the PRD. Road density was generated from road network vector data, and includes all roads in the PRD (highways, national ways, county roads, town roads, etc.). Population density and gross domestic product (GDP) reflect the distribution of the populace and its overall economic health.

#### 2.2.4. Combination of Variables

All variables (see Table 1 for the definitions of these new variables) used in this study were calculated in tiles of about 1 km^2^ resolution, which were mosaicked and windowed to the study area, using ArcGIS 10.3 (ESRI, Redlands, CA, USA). To evaluate how the variable selection method influenced the resulting models, we constructed 3 models based on our original variables: model A, with only climatic variables; model B, with only environmental and socioeconomic variables; and model C, with all climactic, environmental and socioeconomic variables (Table 2).

**Figure 2:**
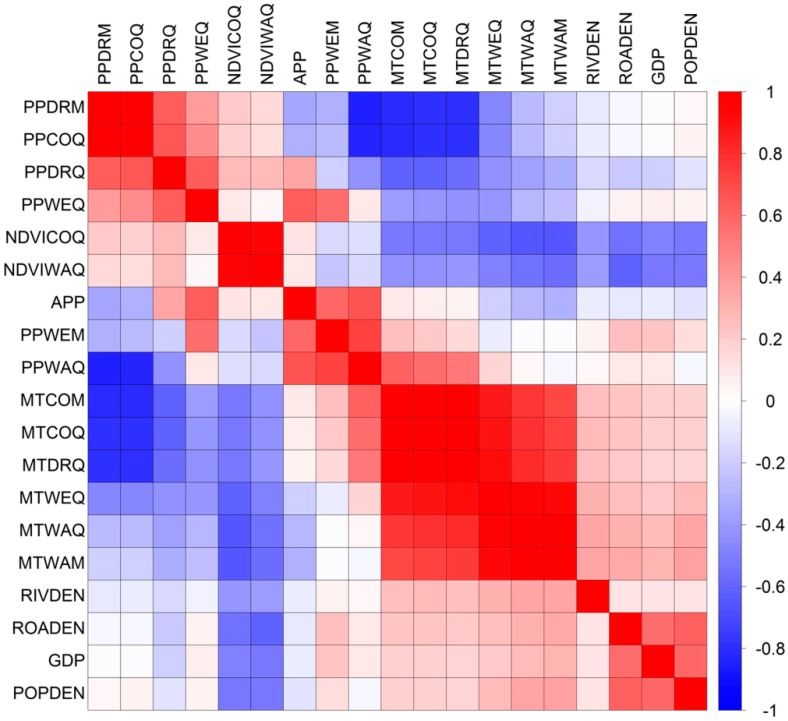
Matrix of correlations between all pairs of original variables.

A high correlation between variables may unjustifiably affect results [25,26] so some studies have integrated highly dependent variables using cluster methods in order to reduce co-linearity [18,27]. In our study, there was a high correlation among some variables, particularly climatic ones (Figure 2). So principal components analysis (PCA) was then used to produce independent predictor variables from our original variables except LUCC variable (Table 2). Principal components with eigenvalues greater than 1 were retained for use as modeling variables. Preliminary results (Table 2) suggested that the first four principal components sufficiently represented 84.6% of the variance of all original continuous variables. The first principal component (F1) represented the majority of the climatic and environmental variables, and the rest of the principal components represented the remainder of the climatic and environmental variables as well as the socioeconomic variables. However, the loadings of climatic variables were generally greater than those of the environmental and socioeconomic variables (Table 2). To reduce the possible weakening of the environmental and socioeconomic variables caused by the integration of principal components, further extraction of climatic principal components was performed as a comparison. Therefore, two PCA models were generated. The first (model D) used the first three principal components (C1–C3) derived from the climatic variables only, as well as the original socioeconomic and environmental variables. The second (model E) used the first four principal components (F1–F4) derived from all original variables, as well as LUCC variable (Table 3).

### 2.3. Modeling and Validation

Ecological Niche Models (ENMs) are able to explore the non-random relationship between disease and environmental factors based on known vectors, hosts, pathogens, and human case information. ENMs achieve fine-scale resolution of distributions limited only by the spatial precision of the input occurrence data and the input environmental datasets, so it can improve the spatial resolution in representing spatial patterns in disease risk [17]. Maxent is one of the most widely used ENM which is a general purpose machine-learning technique based on the principle of maximum entropy. Maxent offers several advantages that make it appropriate for this study: it is non-parametric, requires presence-only data, utilize both continuous and categorical data, incorporates interactions between variables, and produces continuous maps of suitability [28,29]. Maxent has been shown to perform as well as or better than other ENMs [30]. The maximum entropy estimation procedure assumes that the information to be modeled (i.e., DF case distribution) is incomplete and aims to incorporate the minimum amount of non-empirical information [28,29].

We used Maxent model (http://biodiversityinformatics.amnh.org/open_source/maxent/) to generate our ENMs. Empirical DF-case distribution was used to set a number of constraints on the maximum entropy distribution such that the expected value of each predictor variable under this estimated distribution equaled its mean in the empirical distribution [28,29]. The maximum entropy distribution was estimated based on a maximum likelihood approach using a sequential-update algorithm that started from a uniform distribution and sequentially modified one or more weights of the predictor variables to maximize the average log probability of the presence samples [28,29]. Hierarchical maps were obtained with DF risk divided into high, moderate, and low levels. The thresholds of these divisions were set using the “maximum training sensitivity plus specificity logistic threshold” and the “balance training omission, predicted area and threshold value logistic threshold”, which were the empirical thresholds generated by the model [31,32].

In order to evaluate the model results, ten replicates of each model were generated by bootstrapping replicate modeling data where 75% of the DF case points were used for training and the remaining 25% were used for testing. All case points were merged with 10,000 randomly selected background points and were entered into a receiver operating characteristic (ROC) plot analysis to derive the area under the curve (AUC). AUC is a measure of performance that compares the model predictive ability to a random prediction [33]. Model accuracy using AUC was characterized as follows: 0.50–0.60, insufficient; 0.60–0.70, poor; 0.70–0.80, average; 0.80–0.90, good; and 0.90–1.00, excellent [34,35]. In addition, the DF case data in 2014 were used to validate the predicted risk areas by overlaying these DF case points onto the prediction maps.

## 3. Results

### 3.1. Model Performance

All models tested had “good” or “excellent” performance (average AUC > 0.8 in all cases; Table 4). Specifically, models A and C had “excellent” performance (AUC_A_ = 0.904, AUC_C_ = 0.906; Table 4), while models B, E, and D had “good” performance (AUC_D_ = 0.896, AUC_E_ = 0.893; AUC_B_ = 0.882; Table 4). All five models distinguished three levels of risk, but the percentage of the PRD and the percentage of DF cases accounted for at each level of risk varied among the different models (Table 4). Of the models with “excellent” performance in the AUC test, model C identified the smallest amount of the PRD as high risk (8.19%), and accounted for the second largest percentage of DF cases (82.92%; Table 4). Model A, the other model with “excellent” performance, accounted for only the third most cases in the fourth smallest percentage area (Table 4). This evidence suggests that model C is the best predictor of DF risk.

### 3.2. Variable Contributions

**Figure 3:**
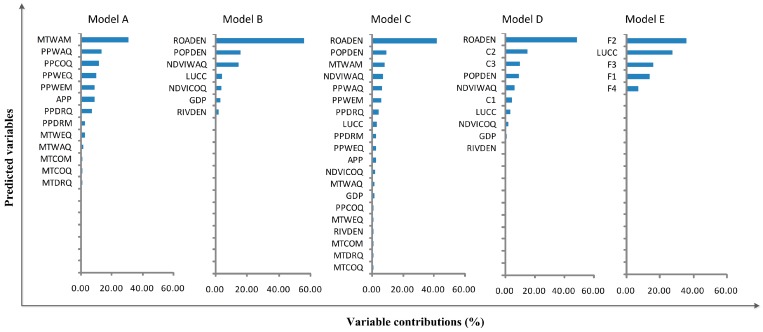
Average contribution of predicted variables over 10 Maxent runs.

In the original-variable models where socioeconomic variables were considered (models B and C), the total contribution of the socioeconomic variables was greater than that of the climatic and environmental variables (up to 55.26%; Figure 3). Similarly, socioeconomic variables also contributed greatly to the first PCA model (model D, 62.57%). In these three models, road density was the most important contributor. In the climactic model (model A), the mean temperature of the warmest month contributed the most. Other important variables are population density (models B, C, and D; contribution >9.00%), the warmest quarter vegetation index (models B, C, D; contribution >6.05%), and the precipitation of the warmest quarter (models A and C; contribution >6.27%; Figure 3).

### 3.3. Risk Responseto Major Predictions

**Figure 4:**
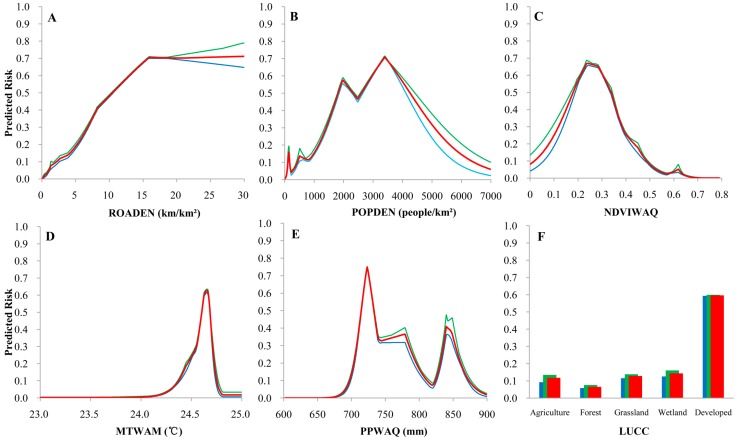
Response curves for the variables in model C as related to the predicted risk of Dengue Fever outbreak. Red line/bar indicates the mean values for the 10 Maxent runs; green line/bar indicates the maximum values; and blue line/bar indicates the minimum value. (**A**) road density; (**B**) population density; (**C**) normalized difference vegetation index (NDVI) of the warmest quarter; (**D**) mean temperature of the warmest month; (**E**) precipitation of the warmest quarter; (**F**) land use and land cover change (LUCC).

According to the predictions of our preferred model (model C), increased road density increased the risk of a DF epidemic until about 15 km/km^2^, at which point the predicted risk stabilized at ~70% (Figure 4A). Increasing population density also increased potential DF epidemic risk in a wave-like pattern up to a point, with a peak at ~4000 people/km^2^ (risk = ~70%) and a smaller peak at ~2000 people/km^2^ (risk = ~60%; Figure 4B). Risk of a DF epidemic in areas with populations greater than ~4000 people/km^2^ rapidly decreased. Risk of a DF epidemic with respect to mean NDVI of the warmest quarter followed a similar pattern, with a peak at about 0.25, and a subsequent decrease (Figure 4C). The mean temperature of the warmest month indicates a sharp peak in DF risk when the warmest month is between 24 °C and 25 °C (Figure 4D). Precipitation in the warmest quarter has a complex relationship to epidemic risk with peak of ~70% at 720 mm, but a low of ~10% at 830 mm (Figure 4E). Developed areas were strongly associated with the DF-epidemic risk, while other types of land use are relatively low risk (Figure 4F).

### 3.4. Spatial Patterns of DF-Epidemic Risk

**Figure 5:**
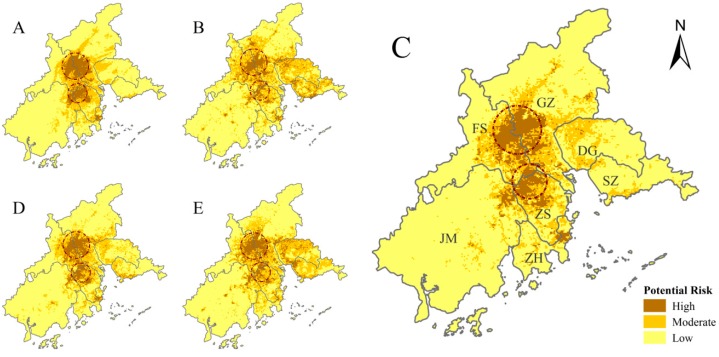
Areas of Dengue Fever outbreak risk, as calculated by the different models (where the letters **A**–**E** relate to the aggregated results from each model). Model definitions are found in Table 3. Model C is the model that we believe best fits the data. Dotted circles indicate areas at highest risk, as chosen by all models.

All of the models that we generated show broadly similar spatial patterns of DF-epidemic risk. The pattern of our preferred model (model C; Figure 5C) is relatively concentrated and contiguous (as in the climactic model A; Figure 5A) while also being discrete and detailed (as in the environmental/socioeconomic model B; Figure 5B). The PCA models D and E were consistent with the overall pattern depicted by model C (Figure 5). They all suggest two areas at high risk for DF epidemics. The first of these is found in the central PRD, on the border of the cities Guangzhou and Foshan (Figure 5C). The other is found in the northern Zhongshan, which is located in the south-central PRD (Figure 5C).

## 4. Discussion

**Figure 6:**
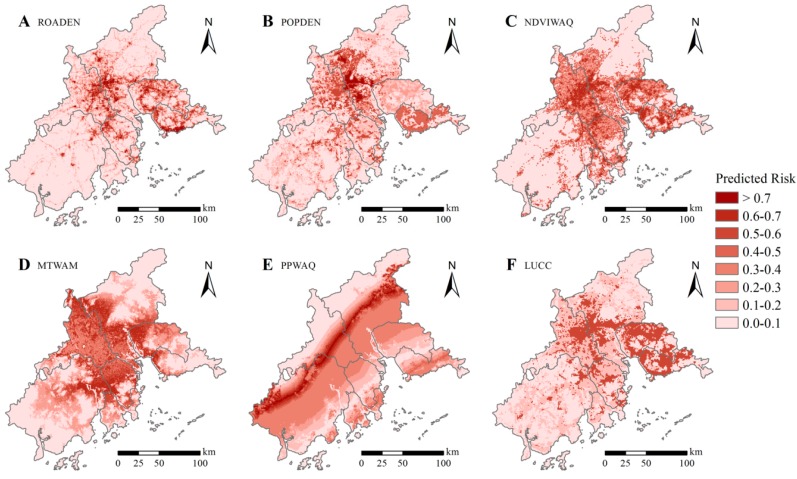
Spatial distribution of the variables related to predicted risk of Dengue Fever: (**A**) road density; (**B**) population density; (**C**) normalized difference vegetation index (NDVI) of the warmest quarter; (**D**) mean temperature of the warmest month; (**E**) precipitation of the warmest quarter; (**F**) land use and land cover change (LUCC).

In this study, we produced five ecological niche models to identify areas in the PRD at high potential risk of a DF epidemic. We also examined a variety of predictive variables that affect DF-epidemic risk based on previously recorded cases locations and other climatic, environmental, and socioeconomic factors. Our models indicate that areas at highest risk for a DF epidemic are distributed along the border between Guangzhou and Foshan (Figure 5). Indeed, over the last 15 years, about 85% of all DF cases in the PRD have been recorded in the Guangzhou/Foshan area; this area is known to be a DF hotspot [8,23]. The use of a fine spatial scale (1 km × 1 km; roughly equivalent to a small township or street), as in our study, is a critical step towards the precise identification of the spatial patterns of DF-epidemic risk in the PRD. At this small scale, we found socioeconomic factors to be the most influential, consistent with other fine-scale studies [11,23,36,37]. In contrast, studies of DF epidemic risk at larger scales (regional, municipal, or county), have found climatic variables to be more important [13,14,15,18]. This discrepancy may be due to the fact that, at this fine spatial scale, socioeconomic differences are obvious, as climatic factors do not vary. At larger scales, differences in climate overwhelm socioeconomic differences (Figure 6). Therefore, we suggest that a more accurate and realistic prediction of the fine-scale spatial distribution of DF-epidemic risk should be primarily based on socioeconomic variables.

In highly urban areas such as the PRD, well-developed road networks facilitate the movement of the population, while high population density increases the probability of mosquito bites. These conditions are ideal for a DF epidemic [9,21,37,38]. In this study, we show that DF epidemic risk in the PRD increases as road and population density increase, up to 15 km/km^2^ and approximately 4000 people/km^2^ (Figure 3). Such densities are typical of ‘urban villages’, under-developed areas within larger cities that are characterized by large, transient populations and poor sanitation [39]. This finding is consistent with work on DF epidemics in Vietnam, where Schmidt et al. identified a narrow range of population densities (~3000 to 7000 people/km^2^), typical of villages and peri-urban areas, which were especially prone to DF outbreaks [40]. Our findings are also consistent with observational data, which show that areas with these critical road and population densities, as found in Guangzhou and Foshan, are the location of the most widespread DF epidemics in the PRD and the Guangdong Province as a whole [7].

In addition to socioeconomic factors, environmental variables, particularly the amount of live vegetation and the mean temperature of the warmest month, also affect the spatial pattern of DF epidemic risk in the PRD (Figure 4). DF epidemic risk is highest when the NDVI of the warmest quarter is 0.2 to 0.4, because the relatively sparse vegetation cover both provides a cooler habitat for the mosquito and serves to retain rainwater, increasing the available mosquito larval habitat [41]. A warmest month with a mean temperature between 24 °C and 25 °C also substantially increases DF epidemic risk, as has been found by others [42,43]. That is, warmer temperatures may shorten the extrinsic incubation period of DF as well as the gonotrophic cycle of mosquito, potentially leading to higher transmission rates [44]. While the warmest month in the PRD (July) is earlier than most DF outbreaks (August to October), this discrepancy can be explained by hysteresis, where the effects of a given stimulus lag behind the occurrence of the stimulus [45].

Our study has certain limitations worthy of discussion and further work. First, we focused on the risk of DF in a static environment, without taking into account the temporal and spatial changes in DF epidemics under dynamic environmental conditions, such as climate change, land coverage and utilization change, population increase, and city expansion. Second, we did not include imported DF cases in our models, even though imported cases typically trigger DF epidemics in the PRD, and may be a critical factor affecting their spatial distribution. Therefore, further study should focus on dynamic characteristics affecting spatial patterns of DF epidemics, and account for the influence of imported DF cases.

## 5. Conclusions

In this study, we have shown that ENMs offer effective tools for the identification of areas at high risk for a DF outbreak. Our work indicates that road and population density, two socioeconomic factors, are critical to the fine-scale spatial pattern of DF epidemic risk. Thus, we have provided a new approach for health agencies in identifying epidemiological risk area in the PRD and southern China. Our results underscore the importance of using a fine scale grid to accurately pinpoint high-risk areas for DF epidemics, and consequently for effective deployment of epidemic monitoring systems.

## Figures and Tables

**Table 1:** Predictor variables used in this study.

Abbreviation	Description	Type
*Climatic Variables*		
MTWAM	Mean temperature of the warmest month (°C)	Continuous
MTCOM	Mean temperature of the coldest month (°C)	Continuous
MTWEQ	Mean temperature of the wettest quarter (°C)	Continuous
MTDRQ	Mean temperature of the driest quarter (°C)	Continuous
MTWAQ	Mean temperature of the warmest quarter (°C)	Continuous
MTCOQ	Mean temperature of the coldest quarter (°C)	Continuous
APP	Annual precipitation (mm)	Continuous
PPWEM	Precipitation in the wettest month (mm)	Continuous
PPDRM	Precipitation in the driest month (mm)	Continuous
PPWEQ	Precipitation in the wettest quarter (mm)	Continuous
PPDRQ	Precipitation in the driest quarter (mm)	Continuous
PPWAQ	Precipitation in the warmest quarter (mm)	Continuous
PPCOQ	Precipitation in the coldest quarter (mm)	Continuous
*Environmental Variables*		
NDVIWAQ	Normalized difference vegetation index (NDVI) of the warmest quarter	Continuous
NDVICOQ	Normalized difference vegetation index (NDVI) of the coldest quarter	Continuous
RIVDEN	River density (km/km^2^)	Continuous
*Socioeconomic Variables*		
ROADEN	Road density (km/km^2^)	Continuous
GDP	Gross domestic product (CNY)	Continuous
POPDEN	Population density (people/km^2^)	Continuous
LUCC	Land use and land cover change (LUCC)	Categorical

**Table 2:** Standardized principal components analysis, using (**a**) climatic variables only, and (**b**) climatic, environmental, and socioeconomic variables. Only the first five principal components are shown in both cases. The variables with the highest absolute loading values are highlighted.

	a. Full Principal Components					b. Climatic Principal Components				
	F1	F2	F3	F4	F5	C1	C2	C3	C4	C5
Eigenvalue	8.185	3.618	2.842	1.427	0.941	7.212	3.245	1.717	0.744	0.063
variance	43.079	19.043	14.958	7.512	4.951	55.48	24.962	13.204	5.722	0.486
Cumulative	43.079	62.122	77.08	84.592	89.544	55.48	80.443	93.647	99.369	99.855
**Variable Loadings**										
MTWAM	0.786	0.446	−0.114	0.329	0.154	0.705	−0.502	0.488	−0.103	−0.013
MTCOM	0.962	−0.207	−0.079	0.101	0.090	0.991	0.038	0.096	0.042	0.055
MTWEQ	0.888	0.211	−0.238	0.283	0.159	0.864	−0.392	0.292	0.112	−0.047
MTDRQ	0.955	−0.173	−0.155	0.134	0.105	0.988	−0.034	0.085	0.121	0.011
MTWAQ	0.829	0.380	−0.146	0.316	0.156	0.763	−0.471	0.438	−0.050	−0.010
MTCOQ	0.964	−0.181	−0.112	0.113	0.095	0.992	−0.003	0.094	0.066	0.033
APP	−0.036	−0.660	0.603	0.338	0.087	0.019	0.883	0.307	0.344	−0.083
PPWEM	0.231	−0.422	0.742	0.018	−0.045	0.211	0.754	0.344	−0.507	0.108
PPDRM	−0.712	0.658	0.071	0.150	0.032	−0.823	−0.437	0.321	−0.154	0.029
PPWEQ	−0.404	−0.052	0.779	0.426	0.089	−0.474	0.520	0.694	−0.093	−0.114
PPDRQ	−0.652	0.168	0.261	0.522	0.205	−0.690	0.058	0.482	0.516	0.151
PPWAQ	0.505	−0.769	0.375	−0.090	−0.050	0.587	0.802	−0.079	−0.068	0.034
PPCOQ	−0.709	0.647	0.119	0.196	0.039	−0.824	−0.401	0.375	−0.137	0.009
NDVIWAQ	−0.629	−0.456	−0.420	0.059	0.243					
NDVICOQ	−0.700	−0.452	−0.318	−0.027	0.197					
RIVDEN	0.349	0.220	0.060	0.308	−0.782					
ROADEN	0.402	0.418	0.500	−0.358	0.098					
GDP	0.353	0.390	0.489	−0.390	0.115					
POPDEN	0.355	0.501	0.436	−0.285	0.204					

**Table 3:** Description of models integrating various types of variables.

Model	Description of Models	Number of Variables
A	Original climatic variables	13
B	Original environmental and socioeconomic variables	7
C	Full original variables	20
D	Climatic principle components (C1, C2, C3) plus original environmental and socioeconomic variables	10
E	Full principle components (F1, F2, F3, F4) plus LUCC variable	5

**Table 4:** Model validation results.

Model	Mean AUC Value		DF Cases in Given Risk Tiers (% of Cases/% Area of the PRD)		
	Training AUC	Test AUC	Low	Moderate	High
A	0.907	0.904	5.13/73.47	12.02/17.92	82.92/8.61
B	0.883	0.882	2.25/64.45	16.01/26.08	81.74/9.47
C	0.910	0.906	3.42/75.85	13.22/15.96	83.35/8.19
D	0.900	0.896	3.80/73.75	12.30/17.88	83.89/8.36
E	0.896	0.893	3.88/65.62	14.77/23.86	81.35/10.51
